# Comparative Study on Growth Performance and Meat Production Traits of Reciprocal Crosses Between Guizhou Recessive White Chickens and Qiandongnan Xiaoxiang Chickens

**DOI:** 10.3390/ani15223262

**Published:** 2025-11-11

**Authors:** Yingping Tian, Xiaoya Wang, Yong Yue, Muhammad Arif, Yaozhou Jiang, Qinsong Liu, Yun Du, Xudong Zhao, Fuping Zhang

**Affiliations:** 1 College of Animal Science, Guizhou University, Guiyang 550025, China; tianyingping394@gmail.com (Y.T.);; 2 Key Laboratory of Animal Genetics, Breeding and Reproduction in the Plateau Mountainous Region, Ministry of Education, Guizhou University, Guiyang 550025, China; 3 Qianxi City Agriculture and Rural Bureau, Qianxi 551599, China; 4 School of Animal Technology and Innovation, Institute of Agricultural Technology, Suranaree University of Technology, Nakhon Ratchasima 30000, Thailand; yongyue1992@126.com; 5 College of Agriculture, Guizhou University, Guiyang 550025, China; arifbiotech144@gmail.com

**Keywords:** reciprocal crosses, Guizhou recessive white chickens, Qiandongnan Xiaoxiang chickens, percentage of heterosis, model fitting, growth performance, meat production traits

## Abstract

**Simple Summary:**

This research investigated whether crossing a local breed from Qiandongnan, China, with a larger fast-growing breed from Guizhou could produce chickens with faster growth rates and higher meat production, thus being more profitable for farmers. We bred different groups of purebred and crossbred chickens and measured their growth, body size, carcass yield and meat quality. The results show that the choice of sire plays a crucial role. Offspring sired by the Guizhou breed grew faster and larger, yielding more meat compared with those sired by the local breed. These crossbred chickens showed a strong heterosis (hybrid vigor), outperforming both parent breeds. Although the pure local chickens had more tender meat, the crossbred chickens from the Guizhou sire offered the best overall combination of growth and meat production. This study provides farmers with a practical and effective strategy: using the Guizhou recessive white as a sire breed can significantly improve the productivity of local chickens, enhancing the efficiency and sustainability of local poultry farming.

**Abstract:**

Indigenous chicken breeds often exhibit desirable meat quality but slower growth. This study evaluated growth, body size, slaughter traits, meat quality, and heterosis in reciprocal crosses between Guizhou recessive white (GW) and Qiandongnan Xiaoxiang (QX) chickens. A complete diallel cross produced four populations (WW: GW♂ × GW♀; QQ: QX♂ × QX♀; QW: QX♂ × GW♀; WQ: GW♂ × QX♀). To assess growth dynamics, body weight was recorded from hatch to 18 weeks and fitted with Logistic, Gompertz, and Von Bertalanffy models. At 18 weeks, 160 birds (40 per group, equal sex ratio) were assessed for body size, carcass yield, and meat quality. The results showed clear paternal effects. For instance, WQ (GW sire) outperformed QW (QX sire): WQ roosters had higher body weight at 18 weeks (1784.1 g vs. QW, *p* < 0.05) and greater heterosis (12.38%, 95%CI: 9.15–15.61 vs. 2.54%, 95%CI: −0.66–5.74). WQ hens also showed stronger heterosis despite similar body weight to QW hens (8.05%, 95%CI: 5.04–11.04 vs. 4.05%, 95%CI: 0.67–7.43). Growth curves were generally best described by the Von Bertalanffy model (R^2^ ≥ 0.998), except in QW roosters, where the Gompertz model fitted better. Hybrid progeny (WQ and QW) showed improved slaughter traits over QQ, with WQ roosters exhibiting higher heterosis rates (14.09–30.71%) than QW (1.08–21.93%). Meat tenderness was superior in QQ, while QW showed advantages over WQ in tenderness and water retention. Overall, crossbreeding enhanced growth and carcass traits, and using GW as the male parent (WQ) was most effective. These findings provide practical evidence for improving Qiandongnan Xiaoxiang chickens through crossbreeding. Moreover, the observed paternal effects on growth traits suggest the need for further investigation into underlying mechanisms such as genomic imprinting and growth-related hormonal pathways.

## 1. Introduction

The most widely consumed foods of animal origin worldwide are poultry meat and eggs, which are not restricted by cultural, traditional, or religious factors [1]. Chicken meat is characterized by its high protein content, low caloric content, low cholesterol levels, and affordability, contributing to its dominance in global meat consumption [2]. The widespread consumption of poultry meat highlights the importance of growth traits, slaughter performance, and meat quality as key targets in poultry breeding programs. In recent years, although commercial chicken breeds have dominated the commercial poultry market in China, indigenous breeds remain prevalent in rural areas and play a vital role in local economic development. Compared to commercial broilers, native chickens exhibit superior disease resistance, enhanced meat quality, and higher nutritional value, making them highly favored by consumers in China and other Asian countries [3,4]. However, limitations such as slow growth rates, high feed conversion ratio (FCR), smaller body sizes, and lower egg production constrain the large-scale development of native chickens [5], resulting in reduced economic returns for farmers and diminished incentives for poultry production. Therefore, enhancing the growth rate and meat production performance of native chickens holds substantial economic significance.

Although several studies have investigated molecular-assisted markers for poultry breeding, their practical application in the selection and improvement of native chickens remains limited. As a result, crossbreeding has served as the primary approach to enhance production performance. For example, the growth rate and carcass fat content of crossbred chickens were improved when Thai Chee native chickens were crossed with Arbor Acre commercial broilers [6]. Similarly, the Marshall breed has been utilized to enhance the egg-laying traits of Nigerian indigenous chickens [7]. Crossbreeding experiments between the Egyptian indigenous Sinai breed and the exotic Lohmann Brown have also demonstrated that crossbreeding can increase body weight and reproductive performance in native chickens [8]. Crosses between different varieties can generate non-additive genetic effects, producing heterosis and resulting in hybrids with superior performance compared to the parental lines [9]. Nevertheless, in hybridization programs, uncertainty often arises regarding which variety should be used as the sire. To address this, several studies have examined differences between forward and backcrossing [10,11], which are frequently associated with maternal effects and sex-linked inheritance [12]. Maternal effects arise from nutrients, mRNAs, and other substances supplied by the mother, which play a crucial role in embryonic development and trait expression [13]. In contrast, sex-linked genes are located on the sex chromosomes and follow distinct inheritance patterns, leading to differences in gene transmission and phenotypic outcomes [14]. Understanding these mechanisms is essential for determining which breed should serve as the sire in hybridization programs targeting specific traits.

Qiandongnan Xiaoxiang chickens, listed in the Chinese Livestock and Poultry Genetic Resources Catalog, represent an important indigenous genetic resource. They are characterized by strong disease resistance, good adaptability, strong foraging ability, and tolerance to roughage. Moreover, their meat is tender and flavorful, with notable nutritional and functional properties, providing health benefits for postpartum women and individuals recovering from prolonged illness. However, their relatively small body size reduces economic profitability in Xiaoxiang chicken farming [15,16]. Thus, improving their productivity is crucial to enhance their market competitiveness. Guizhou recessive white chickens are characterized by four distinctive traits: high egg production (220 eggs annually), green shanks, slow feathering, and recessive white plumage [17]. They can be used as hybrid materials to enhance the production performance of indigenous chickens. The rear band retains the appearance of indigenous chickens, and the sex can be determined by the speed of the feathers [18,19,20]. Accordingly, Guizhou recessive white chickens are considered to possess high commercial value. The utilization of this genetic resource could transform the long-standing limitations in the production and marketing of indigenous chicken germplasm, thereby supporting both the protection and promotion of native chicken breeds. Based on these advantages, we introduced Guizhou recessive white chicken genetic resources to improve the production performance of Qiandongnan Xiaoxiang chickens.

## 2. Materials and Methods

### 2.1. Exprimental Material

**Figure 1:**
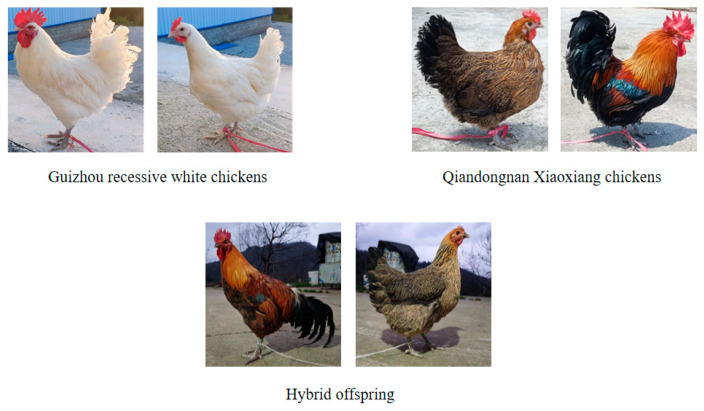
Individual pictures of parents and offspring.

All experimental animals were provided by, and the study was conducted at, the Experimental Poultry Farm of the College of Animal Science, Guizhou University. Four experimental populations were generated through reciprocal crosses of Guizhou recessive white chickens (GW) and Qiandongnan Xiaoxiang chickens (QX): QW (QX males × GW females), WQ (GW males × QX females), WW (GW × GW), and QQ (QX × QX). For the implementation of artificial insemination (AI), fresh semen was first diluted with normal saline in a 1:1 ratio, and subsequent insemination procedures were carried out at four-day intervals. The pictures of the parent and offspring individuals are shown in Figure 1.

Birds were housed in three-tier A-frame cages (each measuring 30 × 40 × 40 cm) with ad libitum access to water, under controlled temperature and uniform environmental conditions. Immunization and feeding management followed the standard protocols of the Guizhou University research farm. Each chick was individually identified with a numbered wing tag immediately after hatching. The experimental period was divided into two phases: the brooding phase (0–6 weeks; birds were housed at 4 per cage) and the growing phase (7–18 weeks; birds were housed at 1 per cage). During the brooding phase, chicks were provided a diet containing 21% crude protein and 2798 kcal/kg metabolizable energy (ME). In the growing phase, the diet was adjusted to 18% crude protein and 3057 kcal/kg ME. The initial brooding temperature was maintained at 35–36 °C and was reduced by approximately 2 °C per week until reaching ambient temperature (20–25 °C). For chicks aged 1–3 days, continuous lighting was provided. From days 4 to 7, photoperiod was reduced to 18–20 h/day, followed by a further reduction of 1 h per day until the birds were fully adapted to natural light conditions.

### 2.2. Body Weight Measurement

To collect body weight data from birds aged 0 to 18 weeks across the four trial groups, measurements were conducted at two–week intervals to minimize stress associated with repeated fasting and weighing. A total of 400 birds were selected (100 per group), and data collection was random each time. All birds were deprived of feed for 12 h before weighing. Body weight was measured in the morning at consistent time points using a digital scale.

### 2.3. Measurement of Body Size and Slaughter Indicators

At 18 weeks of age, 20 males and 20 females were randomly selected from each group, yielding a total of 160 individuals. Following an overnight fast of 12 h, body weight and body measurements were recorded. Birds were humanely stunned and slaughtered by neck bleeding, followed by wet plucking, and slaughter traits were recorded. The slaughtering method complied with the animal welfare guidelines approved by the Guizhou University Subcommittee of Experimental Animal Ethics. The procedures adhered to the nomenclature and calculation methods for poultry productive performance as defined in the Chinese national agricultural standard NY/T 823-2020 [21]. The specific measurement parameters are presented in Table 1.

### 2.4. Meat Quality Measurement

The pH in breast and leg muscles, water loss, shear force, and the cooking loss were measured as part of the analysis. Muscle pH was assessed directly within 45 min post-slaughter using a portable pH meter (SI400, Spectrum Technologies, Delft, The Netherlands). Each sample was measured three times, and the mean value was recorded.

For determination of water loss, samples measuring approximately 2 cm × 2 cm × 1 cm were excised from the breast and leg muscles using surgical clippers. The initial weight (*m*_0_) was recorded, and each sample was placed between 18 layers of qualitative filter paper on both the top and bottom. A meat manometer (Tenovo Meaz-1, Tenovo Food, Beijing, China) was set to 350 N for 5 min, after which the post-pressure weight (*m*_1_) was measured. Water loss was then calculated using the following formula:

$$\mathrm{Water}\;\mathrm{loss}\;(\%)\;=\;\frac{m_{0}-m_{1}}{m_{0}}\times 100.$$

Shear force was determined by cutting muscle samples (4 cm × 1 cm × 1 cm) with a sampler, carefully avoiding fascia and fat. Each sample was tested three times using a Digital Clarion Muscle Tenderness Meter (C-LM3B, Northeast Agricultural University, Harbin, China), and the mean value was calculated.

Cooking loss was assessed by first cutting breast and leg muscle samples into squares weighing approximately 4–5 g (*m*_2_) using surgical scissors to minimize experimental error. Each sample was tied with a thin thread, and a labeled sticky note was attached to identify the sample. The samples were steamed for 30 min in boiling water, then cooled at room temperature for 1 h. The final cooked weight (*m*_3_) was recorded, and cooking loss was calculated using the following formula [22]:

$$\mathrm{Cooking}\;\mathrm{loss}\;(\%)\;=\;\frac{m_{2}-m_{3}}{m_{2}}\times 100$$

### 2.5. Growth Curve Model Fitting

In this study, three nonlinear growth fitting models including Logistic, Gompertz and Von Bertalanffy, were used to fit the body weights of roosters and hens from 0 to 18 weeks of age in four experimental groups. The models and related parameters are shown in Table 2.

### 2.6. Statistics Analysis

Preliminary data organization and screening were conducted using Microsoft Excel 2016 (Microsoft Corp., Redmond, WA, USA) to remove incomplete or missing entries. Normality testing and one-way analysis of variance (ANOVA) were performed using IBM SPSS Statistics 25.0 (IBM Corp., Armonk, NY, USA) to evaluate body weight, body measurements, slaughter traits, and meat quality among the four experimental groups. The Shapiro–Wilk or Kolmogorov–Smirnov tests were used to assess the normality of the data, and Levene’s test was applied to verify homogeneity of variances. When data met the assumptions of normality and homogeneity, one-way ANOVA followed by Tukey’s post hoc test was applied; otherwise, one-way ANOVA followed by the Games–Howell post hoc test was used. Results are presented as mean ± standard deviation (SD).

The model design followed experimental principles: (1) data were stratified by sex to eliminate gender effects; (2) the rearing environment was fully controlled to minimize random effects such as pen differences. Therefore, the final model was defined as “trait value ~ genotype (fixed factor)” without additional covariates, ensuring conciseness and consistency with the experimental logic. Statistical significance was set at *p* < 0.05. Data visualization was performed using GraphPad Prism 8.0.1 (GraphPad Software, San Diego, CA, USA).

The calculation method for Bi-weekly Weight Gain (BWG) was as follows:

$$BWG=\frac{W_{t_{1}}-W_{t_{0}}}{t_{1}-t_{0}}$$

In the formula,

W_t1_—The weight from the prior measurement in grams (g);
W_t0_—The weight from the most recent measurement in grams (g);
t_0_—The age at the time of the prior measurement in days (w);
t_1_—The age at the time of the most recent measurement in days (w);

The percentage of heterosis for the above traits was calculated as the following:

$$H\%=\frac{\bar{F_{1}}-\left(\bar{P_{M}}+\bar{P_{P}}\right)∕2}{\left(\bar{P_{M}}+\bar{P_{P}}\right)∕2}\times 100\%$$

where H% was the percentage of heterosis; $\bar{F_{1}}$, $\bar{P_{M}}$ and $\bar{P_{P}}$ were represented the average phenotypic value of crossbred, maternal lines and paternal lines.

## 3. Results and Discussion

### 3.1. Body Weight

Chickens are generally marketed according to body weight, meaning that increasing live weight directly enhances economic efficiency [23]. According to hybridization theory, crossing varieties with superior traits with those exhibiting inferior traits can leverage genetic recombination and dominant-recessive relationships. This process facilitates the introduction of desirable trait genes into the progeny, thereby enhancing the characteristics of the less favorable varieties and ultimately achieving improved phenotypes [24].

As shown in Table 3, the cumulative body weights of the WW, QQ, WQ, and QW genotypes were compared. At 18 weeks of age, the WW genotype exhibited a body weight of 1884.89 g, which was significantly higher than that of QQ (*p* < 0.05), suggesting that WW has the capacity to improve the weight of QQ. The F1 generations, WQ and QW, also exhibited significantly higher body weights at 18 weeks compared with the parental QQ line (*p* < 0.05). Specifically, WQ roosters reached an average body weight of 1784.1 g, which was significantly greater than that of QW roosters (*p* < 0.05). The heterosis percentage for WQ roosters was 12.38% (95% CI: 9.15–15.61), notably higher than the 2.54% (95% CI: −0.66–5.74) observed in QW roosters. Although no significant difference (*p* > 0.05) was detected between WQ and QW hens at 18 weeks, WQ hens displayed higher heterosis than QW hens. These results indicate that the WQ cross is superior to the QW cross.

From a breeding perspective, the observed hybrid advantage has practical implications for breed conservation and commercial application. Currently, Qiandongnan Xiaoxiang chicken sales rely mainly on purebred stocks, which restricts their protection and sustainable utilization. As shown in Figure 1, the hybrid offspring retained the typical appearance of QQ while exhibiting significantly higher body weights (WQ: rooster 1784 g, hen 1328 g; QQ: rooster 1290 g, hen 1003 g). At a market price of 20 CNY/kg, WQ hybrids yield an additional 494 g in roosters and 325 g in hens, corresponding to increased profits of approximately 9.88 CNY and 6.50 CNY per bird, respectively. This hybridization strategy not only improves farmers’ economic returns but also promotes the conservation and sustainable utilization of indigenous chicken breeds.

Similar findings have been reported in other studies. For example, Yang et al., [25] used heterosis analysis to compare reciprocal crosses between White Leghorn layers and Beijing-You chickens, demonstrating that Beijing-You chickens performed better as sires in crossbreeding schemes. Likewise, different hybrid combinations show varying combining abilities and heterosis depending on the phenotype evaluated. In reciprocal crosses among Chinese black-bone (CB), Thai native (TN), and Hmong black-bone (HB) chickens, TN roosters crossed with CB hens exhibited the greatest potential for growth and carcass traits, whereas HB chickens were unsuitable for crossbreeding under commercial systems [26]. Collectively, these findings underscore the importance of predicting and identifying superior crossbreeding combinations for the development of high-performing chicken lines.

**Figure 2:**
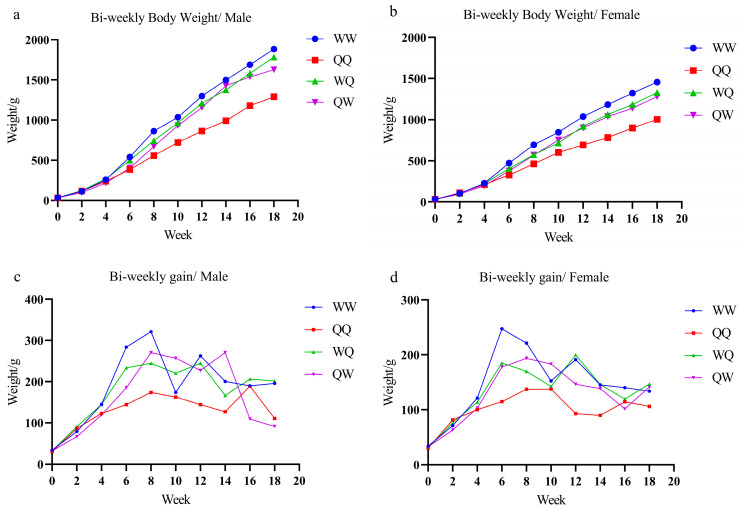
(**a**) Growth curves for roosters from 0–18 weeks across the four experimental groups. (**b**) Growth curves for hens from 0–18 weeks across the four experimental groups. (**c**) Weekly bi-weight gain curves for roosters across the four experimental groups. (**d**) Weekly bi-weight gain curves for hens across the four experimental groups.

Figure 2a,b illustrate the growth and development patterns of the four genotype groups from 0 to 18 weeks. Both WQ and QW genotypes exhibited higher means in body weight compared with QQ, indicating that crossbreeding significantly enhanced body weight in the progeny. This trend is consistent with reports of Xichuan Black-bone chickens improving the body weight of Tengchong Snow chickens [27]. Figure 2c shows the progressive increase in bi-weekly weight gain among the four groups of genotyped roosters from 0 to 8 weeks, peaking at week 8. WW and WQ roosters displayed a decline in bi-weekly weight gain between weeks 8 and 10, followed by accelerated growth from weeks 10 to 12, after which a consistent gain pattern was maintained. In contrast, QW roosters exhibited a bi-weekly weight gain trajectory closely resembling that of QQ roosters. Similarly, Figure 2d demonstrates that bi-weekly weight gain in WW and WQ hens increased steadily from 0 to 6 weeks, declined between 6 and 10 weeks, and then rose again from 10 to 12 weeks, after which a stable pattern was observed. Conversely, bi-weekly weight gain in QQ and QW hens continued to decline until after week 8. These observations indicate that the growth pattern of WQ aligns consistently with WW, whereas QW mirrors QQ, suggesting a paternal genetic effect on growth performance. This phenotypic pattern is consistent with literature on genomic imprinting and paternal allele contributions to growth traits [28,29,30,31,32]. For example, paternally imprinted genes such as IGF2 [29,30,31], along with body-weight–associated quantitative trait loci (QTL) [32,33,34,35,36,37], may underlie the paternal influence observed here. Additionally, paternal genetic factors may modulate growth via the GH–IGF-1 axis [38,39,40,41] or thyroid hormone pathways [42,43], which are key regulators of growth and metabolism. While our current study does not provide direct molecular evidence for these mechanisms, the phenotypic data presented here highlight the practical relevance of paternal effects in crossbreeding and warrant further investigation into genomic imprinting, paternal QTL, and hormonal regulation in future work.

### 3.2. Growth Curve Modeling and Parameter Estimation

**Figure 3:**
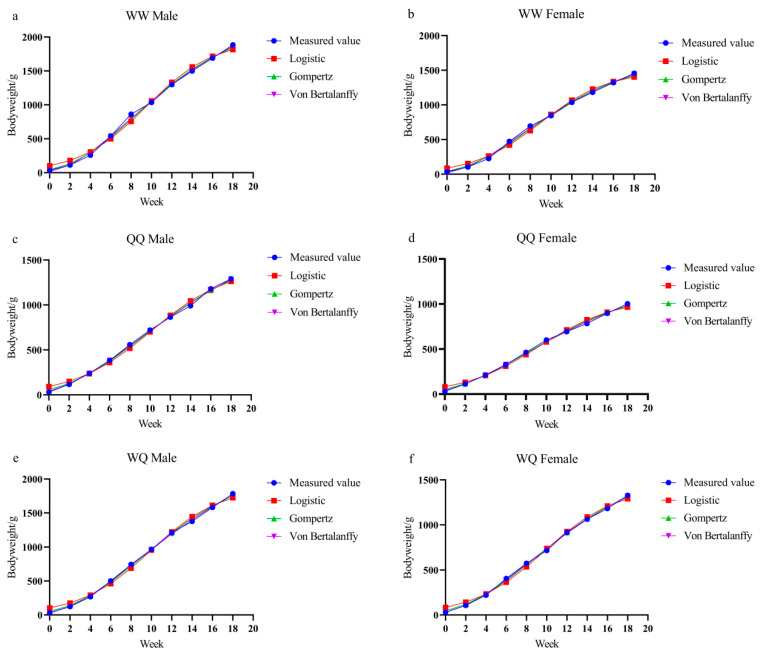

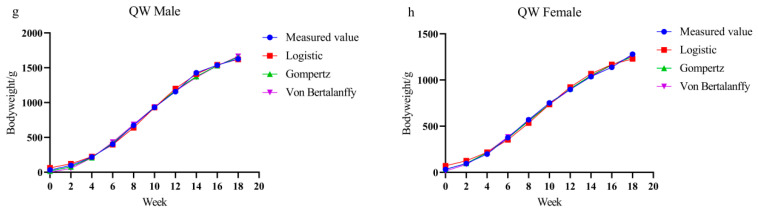
(**a**) Observed body-weight values and nonlinear model fitting curve for WW roosters. (**b**) Observed body-weight values and nonlinear model fitting curve for WW hens. (**c**) Observed body-weight values and nonlinear model fitting curve for QQ roosters. (**d**) Observed body-weight values and nonlinear model fitting curve for QQ hens. (**e**) Observed body-weight values and nonlinear model fitting curve for WQ roosters. (**f**) Observed body-weight values and nonlinear model fitting curve for WQ hens. (**g**) Observed body-weight values and nonlinear model fitting curve for QW roosters. (**h**) Observed body-weight values and nonlinear model fitting curve for QW hens.

Growth curve modeling is an effective approach for elucidating growth relationships. In poultry science, nonlinear models such as Logistic, Gompertz, and Von Bertalanffy are frequently applied to describe the growth patterns of different chicken breeds [44]. These models not only enhance our understanding of growth dynamics but also support the design of precise feeding strategies that reduce feed costs. In the present study, three nonlinear models were employed to analyze the predicted and observed body weights of the WW, QQ, WQ, and QW genotypes from 0 to 18 weeks of age (Figure 3). The results showed that the Logistic, Gompertz, and Von Bertalanffy models all provided an excellent fit to the growth and development patterns of both sexes across the four genotypes. Table 4 presents the goodness-of-fit statistics, with coefficients of determination (R^2^) exceeding 0.990. In a study on the growth of the Turkish native breed Atak-S [45], the Logistic, Gompertz, and Von Bertalanffy models achieved R^2^ values of 0.983–0.997 for both sexes, which aligns well with our results (R^2^ > 0.990). This close similarity made it difficult to identify a single best-fitting model. Therefore, as Akaike’s Information Criterion (AIC) and Bayesian Information Criterion (BIC) have been widely applied to determine optimal growth models in chickens [46,47,48], we also employed AIC and BIC to further distinguish model fit. In general, the closer R^2^ is to 1, the better the fit, whereas smaller AIC and BIC values indicate superior model performance [49]. Only QW roosters fit best to the Gompertz model (AIC: 97.8, BIC: 99.0), whereas all other genotypes were most accurately described by the Von Bertalanffy model, which also predicted the highest mature body weight.

Management practices and nutrient composition can be optimized according to the inflection point age and inflection point weight predicted by the Von Bertalanffy model, thereby enabling each genotype to reach its upper growth limit. As shown in Table 4, the inflection point weight of hens was consistently lower, and their inflection point age earlier, than those of roosters, reflecting sexual dimorphism in growth patterns [50]. Males generally allocate more resources to rapid body weight gain, achieving higher asymptotic body weights, while females direct more resources toward reproductive organ development and preparation for egg laying. Sex hormones play a key role in these dynamics: testosterone promotes muscle development and rapid growth in males [51,52], whereas estrogen in females is associated with fat deposition and egg production [53].

Integrating the findings from Figure 2c,d with the growth curve modeling results, the rearing stages of WW and WQ roosters can be divided into three periods: 0–8 weeks, 8–12 weeks, and from 12 weeks to market readiness. For hens, the stages were defined as 0–6 weeks, 6–12 weeks, and from 12 weeks to the pre-laying stage. In contrast, the rearing stages of QQ and QW roosters were classified as 0–8 weeks, 8–14 weeks, and from 14 weeks to market readiness, while the corresponding stages for hens included 0–10 weeks, followed by transition to the pre-laying stage from week 10 onward.

### 3.3. Body Size Traits

Body size traits are critical indicators of poultry growth performance and provide an essential basis for selection and breeding strategies. The skeletal framework plays a central role in supporting muscle development; larger skeletons offer greater structural capacity to accommodate increased muscle mass. Accordingly, larger bone dimensions are positively associated with improved support for meat yield. As a result, both body size and body weight show a positive correlation, making them reliable indicators for identifying high-quality broilers [54]. Table 5 presents the body size traits of the four genotypes: WW, QQ, WQ, and QW. The calculated heterosis percentages for keel length and tibial circumference were negative, indicating that crossbreeding did not improve these particular traits. However, the objective of this study was to enhance the performance of QQ chickens through the introduction of WW chickens. In this context, shank length in both males and females of the WQ and QW groups was significantly greater than that of the QQ parental line (*p* < 0.05). The heterosis values for shank length ranged from 3.0% to 7.61%, demonstrating measurable improvements in this trait.

Shank length is particularly important for chickens raised in mountainous environments, as longer shanks facilitate faster movement and improve the birds’ ability to escape from predators under free-range or backyard conditions. Moreover, shank length shows a strong genetic correlation with body weight, making it an indirect selection criterion for improving overall growth [55].

The body slope length and chest depth of WQ chickens were significantly greater than those of QQ and QW chickens (*p* < 0.05), with WQ exhibiting superior heterosis compared to QW. A longer body diagonal and deeper chest typically reflect a larger body frame, which provides more space for muscle deposition. This anatomical advantage enhances both muscle volume and body weight, thereby increasing meat production potential [56]. Supporting this, Table 3 shows that body weight in WQ chickens was significantly higher than in both QW and QQ groups (*p* < 0.05), while Table 6 further demonstrates that chest muscle weight in WQ chickens was also significantly greater (*p* < 0.05).

Pelvic width is another key trait, especially in hens, as a wider pelvis supports ovarian and oviductal development, promotes egg production, and reduces the risks of egg retention and related reproductive disorders [57,58]. In the present study, pelvic width in WQ and QW hens was significantly greater than in QQ hens (*p* < 0.05), with no significant difference compared to WW hens (*p* > 0.05). This finding suggests that reciprocal crossbred progeny may inherit improved reproductive traits. Furthermore, heterosis for pelvic width was higher in WQ hens (12.43%, 95%CI: 7.21–17.65) than in QW hens (7.33%,95%CI: 1.68–12.98%), reinforcing the superior performance of the WQ cross.

### 3.4. Slaughter Performance

Carcass characteristics provide an intuitive measure of poultry meat production capacity, and the quantity of meat directly affects the economic returns of chicken farming [59]. Table 6 presents the slaughter performance of purebred and reciprocal cross individuals at 18 weeks of age. Except for breast muscle weight in QW hens, which did not show a significant increase, the live weight prior to slaughter, dressed weight, half-eviscerated weight with giblet, eviscerated weight, breast and leg muscle weights, percentage half-eviscerated yield with giblet, and percentage leg muscle yield in both WQ and QW were significantly higher than those of QQ (*p* < 0.05). Yang et al., [25] investigated whether hybridization can enhance slaughter performance and meat quality in Beijing-You chickens, and their findings claimed that offspring from crosses between White Leghorn layers and Beijing-You chickens had significantly higher live weight, dressed weight, half-eviscerated weight with giblet, eviscerated weight, breast muscle weight, and leg muscle weight compared with pure Beijing-You chickens. These findings are consistent with our results.

Comparison between WQ and WW roosters revealed no significant differences across all slaughter indicators (*p* > 0.05). However, in WQ hens, half-eviscerated weight with giblet, eviscerated weight, and leg muscle weight were significantly lower than those of WW hens (*p* < 0.05). The hybrid WQ roosters exhibited higher heterosis percentages (H%: 14.09–30.71) for all slaughter performance traits compared to QW (H%: 1.08–21.93). This suggests that superior paternal genes from WW, which influence growth rate and meat production, exert strong additive or dominant effects [60], while maternal contributions from QQ may provide additional resources favoring embryonic development [61]. Furthermore, the influence of sex chromosomes and genomic imprinting may reinforce the performance advantages observed in WQ offspring [12].

Overall, these results indicate that the WQ cross more effectively expresses heterosis than QW. Consequently, selecting WW as the sire in QQ breeding programs is recommended to maximize growth performance and meat production in the progeny.

### 3.5. Meat Quality

Indigenous chickens are preferred by consumers over commercial broilers due to attributes such as meat tenderness and freshness [62]. Meat quality is typically assessed using indicators including pH, shear force, cooking loss, and water loss. The pH value reflects the rate of glycogen degradation in muscle post-slaughter [63]. Muscle tenderness is primarily perceived during chewing, with lower shear force indicating more tender meat [64]. Cooking loss and water loss are indicative of muscle juiciness, with lower values reflecting better water retention and higher meat quality [65].

Table 7 presents the effects of hybridization on pH, shear force, cooking loss, and water loss. The pH of WQ progeny did not differ significantly from the parental QQ (*p* > 0.05), whereas QW progeny exhibited a significant decrease in pH (*p* < 0.05), which may be attributed to large slaughter volumes, delayed measurements, and biased glycogen degradation. QQ chickens had lower breast and leg muscle shear values than WW, indicating superior tenderness. Both WQ and QW progeny showed some improvement in tenderness relative to WW, though the differences were mostly not significant, except for the breast muscle shear of QW, which was significantly lower than that of WW (*p* < 0.05).

Pressure-induced water loss in WQ roosters was significantly higher (*p* < 0.05) in both breast and leg muscles, suggesting a reduction in water retention; however, no significant differences (*p* > 0.05) were observed among females across the four genotypes. Conversely, QW progeny exhibited significantly lower cooking loss (*p* < 0.05) in both breast and leg muscles compared with WW, QQ, and WQ groups. Overall, meat quality results indicate that QW crossbred lines outperform WQ in terms of balancing tenderness and water retention.

Crossbreeding (WQ, QW) positively influenced growth performance and meat production. At 18 weeks, crossbred progeny exhibited significantly higher body weights than the parental QQ line (*p* < 0.05), with WQ roosters reaching 1784 g compared with 1290 g in QQ roosters. Crossbreds also showed improved slaughter traits, including higher dressed weight, eviscerated weight, and breast and leg muscle weights, resulting in increased overall meat yield. Notably, these enhancements in growth and carcass traits were achieved without adverse effects on meat quality, as parameters such as tenderness, water-holding capacity, and pH remained comparable to parental lines. These findings suggest that crossbreeding WQ and QW with QQ not only enhances production performance but also maintains product quality, supporting the practical application of crossbreeding strategies to improve the efficiency and economic value of indigenous chicken breeds.

### 3.6. Application Strategies for Scaling the WQ Hybrid in Local Poultry Industry

This study provides the first confirmation that crossbreeding with Guizhou Recessive White chickens (WW) enhances the growth and meat production performance of Qiandongnan Xiaoxiang chickens (QQ). Notably, the WQ hybrid combination exhibited a significant improvement in 18-week body weight, showing 12.38% heterosis in roosters, while retaining QQ’s distinctive feather coloration and desirable meat quality— key traits valued in local markets.

Building on these findings, the following practical strategies are proposed to facilitate the translation of this hybrid advantage into on-farm application. A three-tier propagation system will ensure stable inheritance of superior traits: (1) a grandparent core population managed via pedigree recording to prevent inbreeding; (2) a parent generation optimized for fertility (≥92% via artificial insemination) and standardized hatching; (3) commercial flocks supplied to local farms for consistent production.

Supported by the “company + farmer” model (including chick supply, technical training, and market guarantees) and government subsidies for breeding equipment, this approach will help unlock the production potential of the WQ hybrid, contributing to the sustainable development of the Qiandongnan Xiaoxiang Chicken industry.

## 4. Conclusions

This study investigated the effects of reciprocal crosses *between* Guizhou Recessive White (GW) and Qiandongnan Xiaoxiang (QQ) chickens on growth performance and meat production traits. The WQ cross combination (GW sire × QQ dam) exhibited the best performance in growth and carcass traits, significantly outperforming the QQ and QW genotypes (*p* < 0.05). Based on heterosis analysis, a breeding line derived from the WQ combination could be established for practical poultry production. Analysis of growth patterns further revealed that WQ progeny followed the growth trajectory of the WW parental line, whereas QW progeny mirrored the QQ line, suggesting a potential paternal influence on growth and development. Future studies should investigate whether paternal inheritance regulates offspring growth through mechanisms such as genomic imprinting or growth hormone-related pathways, as understanding these processes may provide valuable insights for optimizing crossbreeding strategies in local chicken breeds.

## Figures and Tables

**Table 1:** Detailed measurements of body size and slaughter indicators.

Measurement Index	Measurement Method
Body Slope Length (BSL)/cm	Measured as the distance from the shoulder joint to the sciatic tuberosity using a tape measure.
Keel Length (KL)/cm	Measured as the distance from the anterior tip to the posterior end of the keel using a tape measure.
Chest Depth (CD)/mm	Measured as the vertical distance from the first thoracic vertebra to the anterior edge of the keel using calipers.
Chest Width (CW)/mm	Measured as the distance between the left and right shoulder joints using calipers.
Pelvis Width (PW)/mm	Measured as the distance between the left and right iliac tuberosities using calipers.
Shank Length (SL)/mm	Measured as the distance from the hock joint to the tip of the third toe using calipers.
Shin Girth (SG)/cm	Measured as the circumference at the midpoint of the shank using a tape measure.
Live Weight Before Slaughter (LW)/g	Live weight measured after a 12 h fast prior to slaughter.
Dressed Weight (DW)/g	Weight after exsanguination, defeathering, and removal of claw sheaths and beak sheaths.
Half-eviscerated Weight with Giblet (HEW)/g	Carcass weight after removal of the trachea, esophagus, crop, gastrointestinal tract, spleen, pancreas, gallbladder, reproductive organs, and stomach contents.
Eviscerated Weight (EW)/g	Calculated as half-eviscerated weight with giblets minus the heart, liver, proventriculus, gizzard, lungs, abdominal fat, head, and feet.
Breast Muscle Weight (BMW)/g	Weight of the pectoral muscles completely removed from both sides of the keel using a scalpel.
Leg Muscle Weight (LMW)/g	Weight of thigh and drumstick muscles after removal of skin, bones, and fat.
Dressed Percentage (DP)	(DW/LW) × 100%
Percentage Half-eviscerated Yield with Giblet (PHE)	(HEW/LW) × 100%
Percentage of Eviscerated Yield (PE)	(EW/LW) × 100%
Percentage of Breast Muscle Yield (PBM)	(BMW/EW) × 100%
Percentage of Leg Muscle Yield (PLM)	(LMW/EW) × 100%

**Table 2:** The mathematical expression of fitted model.

Models	Formula	Inflection Point Weight	Inflection Point Weekly Age
Logistic	Y = A/(1 + Be^−kt^)	A/2	(ln B)/k
Gompertz	Y = Ae ^−Bexp(−kt)^	A/e	(ln B)/k
VonBertalanffy	Y = A (1 − Be^−kt^)^3^	8A/27	(ln 3B)/k

Y is body weight at t weeks of age; A is the growth limit; B is the constant scale; exp is the natural expo-nential function; k is the growth rate constant.

**Table 3:** Comparison of growth weights of Guizhou recessive white chickens, Qiandongnan Xiaoxiang chickens and the progeny of forward and backward crosses.

Week	Gender	Body Weight/g				H%(WQ)/95%CI	H%(QW)/95%CI
		WW	QQ	WQ	QW		
0	Male	33.68 ± 2.47 ^a^	30.47 ± 2.8 ^b^	31.68 ± 3.37 ^b^	31.77 ± 2.22 ^b^	1.23 (−2.92%, 0.46%)	−0.95 (−2.34%, 0.44%)
	Female	33.26 ± 2.33 ^a^	30.19 ± 2.65 ^c^	30.39 ± 3.19 ^bc^	32.11 ± 3.12 ^ab^	4.21 (−6.21%, −2.21%)	1.21 (−0.71%, 3.13%)
2	Male	112.6 ± 18.53 ^b^	116.88 ± 12.64 ^ab^	122.42 ± 14.64 ^a^	98.29 ± 13.36 ^c^	6.69 (4.49%, 8.89%)	−14.34 (−16.60%, −12.08%)
	Female	104.22 ± 15.34 ^b^	111.41 ± 14.36 ^a^	106.52 ± 15.8 ^ab^	95.06 ± 15.69 ^c^	−1.20 (−4.65%, 2.25%)	−11.83 (−15.26%, −8.40%)
4	Male	257.48 ± 43.81 ^a^	239.6 ± 24.17 ^b^	266.81 ± 33.94 ^a^	217.36 ± 38.77 ^b^	7.35 (3.65%, 11.05%)	−12.55 (−16.43%, −8.67%)
	Female	225.3 ± 36.12 ^a^	211.48 ± 28.8 ^ab^	220.24 ± 34.39 ^a^	197.89 ± 23.31 ^b^	0.85 (−2.42%, 4.12%)	−9.39 (−12.29%, −6.49%)
6	Male	541.15 ± 69.73 ^a^	383.64 ± 45.18 ^c^	500.23 ± 62.32 ^b^	402.44 ± 82.83 ^c^	8.18 (4.70%, 11.66%)	−12.97 (−17.28%, −8.66%)
	Female	472.56 ± 64.67 ^a^	326.07 ± 51.71 ^d^	405.28 ± 52.09 ^b^	375.48 ± 32.03 ^c^	1.49 (−1.21%, 4.19%)	−5.97 (−8.40%, −3.54%)
8	Male	862.23 ± 80.78 ^a^	557.55 ± 54.54 ^d^	744.7 ± 97.7 ^b^	673.08 ± 91.8 ^c^	4.90 (1.72%, 8.08%)	−5.19 (−8.26%, −2.10%)
	Female	693.56 ± 76.18 ^a^	463.07 ± 60.96 ^c^	574.78 ± 85.56 ^b^	569.08 ± 68.46 ^b^	−0.61 (−3.71%, 2.49%)	−1.60 (−4.40%, 1.20%)
10	Male	1036.01 ± 107.29 ^a^	720.15 ± 81.78 ^c^	965.17 ± 132.84 ^b^	930.07 ± 110.53 ^b^	9.92 (6.33%, 13.51%)	5.92 (2.71%, 9.13%)
	Female	845.84 ± 76.33 ^a^	600.49 ± 74.63 ^c^	717.08 ± 90.17 ^b^	752.1 ± 94.88 ^b^	−0.84 (−3.82%, 2.14%)	4.00 (0.89%, 7.09%)
12	Male	1298.66 ± 130.69 ^a^	864.16 ± 97.49 ^c^	1209.83 ± 143.61 ^b^	1157.09 ± 126.81 ^b^	11.88 (8.26%, 15.50%)	7.00 (3.63%, 10.37%)
	Female	1037.2 ± 93.15 ^a^	693.29 ± 75.27 ^c^	916.87 ± 109.57 ^b^	898.49 ± 108.21 ^b^	5.97 (2.96%, 8.98%)	3.84 (0.84%, 6.84%)
14	Male	1499.21 ± 165.7 ^a^	990.84 ± 110.45 ^c^	1376.42 ± 135.55 ^b^	1427.75 ± 154.47 ^ab^	10.55 (7.92%, 13.20%)	14.68 (11.78%, 17.58%)
	Female	1182.21 ± 122.85 ^a^	782.95 ± 84.68 ^c^	1062.63 ± 124.77 ^b^	1036.98 ± 134.77 ^b^	8.15 (5.35%, 10.95%)	5.54 (2.58%, 8.50%)
16	Male	1689.1 ± 203.12 ^a^	1179.8 ± 120.44 ^c^	1582.6 ± 181.64 ^b^	1536.85 ± 169.58 ^b^	10.33 (6.99%, 13.67%)	7.14 (3.94%, 10.34%)
	Female	1322.24 ± 149.78 ^a^	897.27 ± 93.76 ^c^	1181.68 ± 141.11 ^b^	1138.41 ± 151.64 ^b^	6.48 (3.54%, 9.42%)	2.58 (−0.52%, 5.68%)
18	Male	1884.89 ± 219.67 ^a^	1290.33 ± 128.98 ^d^	1784.1 ± 182.07 ^b^	1627.94 ± 178.41 ^c^	12.38 (9.15%, 15.61%)	2.54 (−0.66%, 5.74%)
	Female	1455.96 ± 147.47 ^a^	1003.24 ± 101.76 ^c^	1328.57 ± 154.9 ^b^	1279.41 ± 183.01 ^b^	8.05 (5.04%, 11.04%)	4.05 (0.67%, 7.43%)

The superscripts of the letters in the table represent the results of multiple comparisons between genotypes (*p* < 0.05): According to the results of homogeneity of variance tests for each trait, the Tukey test was used when the variances were homogeneous, and the Games–Howell test was used when the variances were heterogeneous. The same letters indicate no significant difference, while different letters indicate significant difference. The same for below. The 95% CI was calculated using the Z-distribution (when n ≥ 30, it follows a Z-distribution; when n < 30, it follows a t-distribution).

**Table 4:** Estimated parameters of fitted models for males and females.

Gender	Model	Parameter Estimates						Inflection Point Weight/g	Inflection Point Weekly Age
		A	B	K	R^2^	AIC	BIC		
WW Male	Logistic	1950.874	18.255	0.306	0.991	117.9	119.1	975.44	9.49
	Gompertz	2258.256	3.949	0.165	0.997	106.4	107.7	830.85	8.32
	Von Bertalanffy	2547.859	0.817	0.117	0.998	102.5	103.7	754.92	7.66
WW Female	Logistic	1491.06	16.217	0.309	0.992	112.0	113.2	745.53	9.02
	Gompertz	1697.253	3.749	0.171	0.998	99.4	100.6	624.45	7.73
	Von Bertalanffy	1884.251	0.792	0.124	0.999	95.1	96.3	558.30	6.98
QQ Male	Logistic	1417.718	14.355	0.264	0.993	107.0	108.2	708.86	10.09
	Gompertz	1736.208	3.425	0.134	0.998	94.4	95.6	638.78	9.19
	Von Bertalanffy	2079.531	0.734	0.089	0.999	87.6	88.8	616.16	8.87
QQ Female	Logistic	1059.915	12.1	0.268	0.992	103.3	104.5	529.96	9.30
	Gompertz	1255.005	3.18	0.142	0.998	91.6	92.8	461.74	8.15
	Von Bertalanffy	1446.659	0.701	0.098	0.999	85.6	86.8	428.64	7.59
WQ Male	Logistic	1893.218	17.486	0.288	0.994	113.4	114.7	946.61	9.94
	Gompertz	2273.604	3.782	0.149	0.999	98.4	99.7	836.50	8.93
	Von Bertalanffy	2669.429	0.784	0.1	0.999	91.0	92.2	790.94	8.55
WQ Female	Logistic	1415.121	15.782	0.283	0.994	106.2	107.4	707.56	9.75
	Gompertz	1692.523	3.593	0.147	0.999	91.8	93.0	622.71	8.70
	Von Bertalanffy	1976.638	0.759	0.1	0.999	86.0	87.2	585.67	8.23
QW Male	Logistic	1706.714	26.144	0.344	0.998	98.7	99.9	853.36	9.49
	Gompertz	1951.742	4.678	0.185	0.999	97.8	99.0	718.08	8.34
	Von Bertalanffy	2167.389	0.923	0.133	0.997	105.3	106.5	642.19	7.66
QW Female	Logistic	1308.517	17.465	0.311	0.995	105.3	106.5	654.26	9.20
	Gompertz	1510.082	3.837	0.168	0.999	89.8	91.0	555.59	8.00
	Von Bertalanffy	1699.367	0.799	0.119	0.999	88.9	90.1	503.52	7.35

AIC: Akaike’s information criterion, BIC: Bayesian information criterion.

**Table 5:** Comparison of body size traits of Guizhou recessive white chickens, Qiandongnan Xiaoxiang chickens and the progeny of forward and backward crosses.

Body-Meter Index/cm	Gender	WW	QQ	WQ	QW	H%(WQ)/95%CI	H%(QW) 95%CI
Body Slope Length	Male	20.81 ± 0.7 ^a^	16.09 ± 0.87 ^d^	19.76 ± 0.97 ^b^	18.33 ± 1.23 ^c^	7.10 (2.35%, 11.85%)	−0.65 (−6.89%, 5.59%)
	Female	18.29 ± 0.86 ^a^	14.7 ± 0.87 ^d^	17.25 ± 0.82 ^b^	16.2 ± 0.77 ^c^	4.29 (0.15%, 8.43%)	−1.51 (−6.18%, 3.16%)
Keel Length	Male	10.87 ± 0.58 ^a^	10.01 ± 0.55 ^b^	10.25 ± 0.9 ^b^	8.84 ± 0.7 ^c^	−1.82 (−9.97%, 6.33%)	−15.33 (−21.98%, −8.68%)
	Female	9.45 ± 0.46 ^a^	9.8 ± 0.72 ^a^	8.75 ± 0.61 ^b^	8.3 ± 0.72 ^b^	−9.47 (−15.82%, −3.12%)	−13.71 (−20.15%, −7.27%)
Chest Width	Male	7.76 ± 0.67 ^a^	6.43 ± 0.41 ^b^	7.89 ± 0.56 ^a^	8.13 ± 0.67 ^a^	11.21 (5.87%, 16.55%)	14.59 (8.21%, 20.97%)
	Female	7.18 ± 0.47 ^a^	6.2 ± 0.41 ^c^	6.58 ± 0.53 ^bc^	6.9 ± 0.72 ^ab^	−1.72 (−8.05%, 4.61%	4.11 (−2.63%, 10.85%)
Chest Depth	Male	10.59 ± 0.53 ^a^	8.37 ± 0.58 ^c^	10.4 ± 0.71 ^a^	9.62 ± 0.43 ^b^	9.70 (3.15%, 16.25%)	1.48 (−2.41%, 5.37%)
	Female	9.18 ± 0.53 ^a^	8.4 ± 0.5 ^b^	9.11 ± 0.42 ^a^	8.6 ± 0.53 ^b^	3.41 (−0.98%, 7.80%)	−2.38 (−7.95%, 3.19%)
Pelvis Width	Male	10.27 ± 0.57 ^a^	6.97 ± 0.57 ^c^	9.68 ± 0.52 ^b^	9.82 ± 0.72 ^ab^	12.30 (6.98%, 17.62%)	13.92 (7.23%, 20.61%)
	Female	8.72 ± 0.67 ^a^	6.9 ± 0.48 ^b^	8.82 ± 0.6 ^a^	8.4 ± 0.63 ^a^	12.43 (7.21%, 17.65%)	7.33 (1.68%, 12.98%)
Shank Length	Male	11.22 ± 0.58 ^a^	8.76 ± 0.5 ^c^	10.34 ± 0.61 ^b^	10.29 ± 0.63 ^b^	3.50 (−2.13%, 9.13%)	3.00 (−2.94%, 8.94%)
	Female	9.32 ± 0.69 ^a^	7.1 ± 0.49 ^c^	8.76 ± 0.7 ^b^	8.8 ± 0.67 ^b^	6.63 (1.52%, 11.74%)	7.61 (2.13%, 13.09%)
Shin Girth	Male	4.1 ± 0.21 ^a^	3.63 ± 0.18 ^b^	3.75 ± 0.25 ^b^	3.02 ± 0.22 ^c^	−2.98 (−6.37%, 0.41%)	−21.86 (−25.19%, −18.53%)
	Female	3.57 ± 0.21 ^a^	3.3 ± 0.22 ^b^	3.3 ± 0.24 ^b^	2.7 ± 0.16 ^c^	−4.76 (−9.23%, −0.29%)	−19.77 (−23.45%, −16.09%)

In the same row, values with no letter or the same letter superscripts mean no significant difference (*p* > 0.05), while values with different small letter superscripts mean significant difference (*p* < 0.05). The 95% CI was calculated using the t-distribution (when n ≥ 30, it follows a Z-distribution; when n < 30, it follows a t-distribution).

**Table 6:** Comparison of Slaughter performance of Guizhou recessive white chickens, Qiandongnan Xiaoxiang chickens and the progeny of forward and backward crosses.

Slaughtering Indicators	Gender	WW	QQ	WQ	QW	H%(WQ)	H%(QW)
Live Weight Before/g Slaughter/g	Male	1894.6 ± 211.22 ^a^	1162.2 ± 101.36 ^c^	1743.7 ± 220.48 ^ab^	1658.97 ± 185.36 ^b^	14.09 (6.45%, 21.73%)	8.54 (1.82%, 15.26%)
	Female	1415.6 ± 135.1 ^a^	1052.55 ± 96.21 ^b^	1299.8 ± 151.25 ^a^	1279.41 ± 183.01 ^a^	5.33 (−1.28%, 11.94%)	3.67 (−3.81%, 11.15%)
Dressed Weight/g	Male	1605.3 ± 202.62 ^a^	1027.2 ± 101.36 ^c^	1491.5 ± 200.73 ^ab^	1433.85 ± 173.54 ^b^	13.31 (5.83%, 20.79%)	8.93 (2.27%, 15.59%)
	Female	1214.7 ± 118.1 ^a^	903.61 ± 105.83 ^b^	1120.0 ± 137.99 ^a^	1110.20 ± 169.66 ^a^	5.75 (−0.71%, 12.21%)	4.82 (−1.53%, 11.17%)
Half-eviscerated Weight with Giblet/g	Male	1411.4 ± 190.1 ^a^	821.56 ± 81.09 ^b^	1309.45 ± 179 ^a^	1292.46 ± 156.29 ^a^	17.28 (9.51%, 25.05%)	15.76 (7.92%, 23.60%)
	Female	1077.9 ± 111.41 ^a^	730.67 ± 86.75 ^c^	961.15 ± 138.98 ^b^	963.13 ± 146.37 ^b^	6.29 (−0.18%, 12.76%)	6.50 (−0.05%, 13.05%)
Eviscerated Weight/g	Male	1159.5 ± 167.41 ^a^	725.47 ± 71.35 ^c^	1080.3 ± 152.87 ^ab^	1039.73 ± 136.07 ^b^	14.62 (7.15%, 22.09%)	10.31 (3.02%, 17.60%)
	Female	887.63 ± 93.55 ^a^	645.48 ± 76.34 ^c^	790.45 ± 112.2 ^b^	770.74 ± 108.23 ^b^	3.12 (−3.35%, 9.59%)	0.55 (−5.80%, 6.90%)
Breast Muscle Weight/g	Male	179.54 ± 38.75 ^a^	116.28 ± 10.87 ^c^	176.1 ± 26.88 ^a^	149.51 ± 22.1 ^b^	19.06 (10.82%, 27.30%)	1.08 (−6.89%, 9.05%)
	Female	151.83 ± 24.77 ^a^	120.26 ± 6.72 ^c^	138.50 ± 22.07 ^ab^	128.70 ± 23.62 ^bc^	1.80 (−5.12%, 8.72%)	−5.40 (−12.09%, 1.29%)
Leg Muscle Weight/g	Male	309.20 ± 56.89 ^a^	154.36 ± 12.17 ^b^	302.95 ± 48.85 ^a^	282.6 ± 41.03 ^a^	30.71 (21.35%, 40.07%)	21.93 (13.02%, 30.84%)
	Female	221.85 ± 28.89 ^a^	115.88 ± 11.12 ^c^	195.20 ± 30.79 ^b^	181.31 ± 28.17 ^b^	15.60 (7.52%, 23.68%)	7.37 (−0.28%, 15.02%)
Dressed Percentage%	Male	84.6 ± 2.72 ^c^	88.3 ± 0.99 ^a^	85.46 ± 2.38 ^bc^	86.35 ± 1.59 ^b^	−1.16 (−3.87%, 1.55%)	−0.13 (−2.81%, 2.55%)
	Female	85.8 ± 1.34	85.66 ± 2.2	86.1 ± 2.51	86.65 ± 1.61	0.48 (−2.01%, 2.97%)	1.07 (−1.40%, 3.54%)
Percentage Half-eviscerated Yield with Giblet%	Male	74.3 ± 3.15 ^b^	70.6 ± 0.79 ^c^	75.02 ± 2.12 ^b^	77.88 ± 3.14 ^a^	3.49 (0.98%, 6.00%)	7.44 (4.87%, 10.01%)
	Female	76.1 ± 2.94 ^a^	69.26 ± 1.89 ^b^	73.8 ± 4.5 ^a^	75.20 ± 2.81 ^a^	1.55 (−0.88%, 3.98%)	3.44 (1.05%, 5.83%)
Percentage of Eviscerated Yield%	Male	61.0 ± 3.05	62.3 ± 0.68	61.86 ± 2.07	62.6 ± 3.21	0.25 (−2.17%, 2.67%)	1.45 (−0.94%, 3.84%)
	Female	62.6 ± 1.76 ^a^	61.18 ± 1.64 ^ab^	60.7 ± 3.35 ^ab^	60.34 ± 3.55 ^b^	−1.95 (−4.28%, 0.38%)	−2.55 (−4.85%, −0.25%)
Percentage of Breast Muscle Yield%	Male	15.3 ± 1.94 ^ab^	16.1 ± 2.01 ^a^	16.3 ± 0.91 ^a^	14.39 ± 1.27 ^b^	3.36 (0.92%, 5.80%)	−8.75 (−11.06%, −6.44%)
	Female	17.0 ± 1.64 ^b^	18.82 ± 1.93 ^a^	17.5 ± 1.62 ^ab^	16.64 ± 1.39 ^b^	−2.15 (−4.43%, 0.13%)	−7.22 (−9.47%, −4.97%)
Percentage of Leg Muscle Yield%	Male	26.6 ± 2.53 ^a^	21.3 ± 0.4 ^b^	28 ± 1.46 ^a^	27.2 ± 2.18 ^a^	16.89 (14.02%, 19.76%)	13.55 (10.75%, 16.35%)
	Female	24.9 ± 1.88 ^a^	18.00 ± 0.4 ^b^	24.7 ± 1.84 ^a^	23.59 ± 2.47 ^a^	14.96 (12.28%, 17.64%)	9.75 (7.12%, 12.38%)

In the same row, values with no letter or the same letter superscripts mean no significant difference (*p* > 0.05), while values with different small letter superscripts mean significant difference (*p* < 0.05). The 95% CI was calculated using the t-distribution (when n ≥ 30, it follows a Z-distribution; when n < 30, it follows a t-distribution).

**Table 7:** Comparison of meat quality of Guizhou recessive white chickens, Qiandongnan Xiaoxiang chickens and the progeny of forward and backward crosses.

Meat Quality Indicators	Gender	Position	WW	QQ	WQ	QW
pH (45 min)	Male	Chest	6.29 ± 0.22 ^a^	5.75 ± 0.16 ^b^	5.82 ± 0.16 ^b^	5.36 ± 0.42 ^c^
		Leg	6.36 ± 0.26 ^a^	6.11 ± 0.22 ^a^	6.12 ± 0.24 ^a^	5.59 ± 0.42 ^b^
	Female	Chest	6.37 ± 0.27 ^a^	5.79 ± 0.16 ^c^	6.1 ± 0.27 ^b^	5.56 ± 0.36 ^c^
		Leg	6.39 ± 0.28 ^a^	6.1 ± 0.21 ^a^	6.15 ± 0.26 ^a^	5.68 ± 0.4 ^b^
Shear force/N	Male	Chest	18.91 ± 4.8 ^a^	16.56 ± 2.91 ^ab^	15.93 ± 6.27 ^ab^	12.85 ± 5.33 ^b^
		Leg	33.03 ± 9.87 ^a^	16.12 ± 2.14 ^b^	32.42 ± 12.23 ^a^	26.45 ± 7.59 ^a^
	Female	Chest	19.00 ± 4.57 ^a^	16.08 ± 2.34 ^ab^	14.22 ± 6.4 ^ab^	13.58 ± 5.4 ^b^
		Leg	27.79 ± 6.81 ^a^	18.19 ± 1.92 ^b^	21.12 ± 9.91 ^ab^	24.27 ± 6.21 ^ab^
Water loss%	Male	Chest	19.00 ± 4.17 ^bc^	16.18 ± 3.2 ^c^	23.00 ± 5.01 ^a^	21.22 ± 2.42 ^ab^
		Leg	19.33 ± 3.87 ^b^	15.77 ± 3.49 ^b^	22.89 ± 3.89 ^a^	17.46 ± 3.6 ^b^
	Female	Chest	20.41 ± 5.27	20.02 ± 5.84	24.51 ± 5.68	22.97 ± 2.32
		Leg	17.44 ± 3.41	19.68 ± 4.17	20.44 ± 4.18	17.92 ± 3.63
Cooking loss%	Male	Chest	37.09 ± 2.69 ^a^	36 ± 2.53 ^a^	37.06 ± 2.58 ^a^	28.71 ± 2.5 ^b^
		Leg	41.74 ± 3.72 ^a^	43.41 ± 2.96 ^a^	43.05 ± 2.53 ^a^	33.06 ± 4.23 ^b^
	Female	Chest	37.03 ± 2.05 ^a^	34.98 ± 4.94 ^a^	36.93 ± 3.17 ^a^	29.49 ± 3.31 ^b^
		Leg	40.15 ± 3.25 ^a^	40.31 ± 4.53 ^a^	42.58 ± 3.83 ^a^	33.89 ± 3.01 ^b^

In the same row, values with no letter or the same letter superscripts mean no significant difference (*p* > 0.05), while values with different small letter superscripts mean significant difference (*p* < 0.05).

## Data Availability

Data will be made available from the corresponding author upon reasonable request.
